# Prognostic Impact of Serum β_2_-Microglobulin Levels in Hodgkin Lymphoma Treated with ABVD or Equivalent Regimens: A Comprehensive Analysis of 915 Patients

**DOI:** 10.3390/cancers16020238

**Published:** 2024-01-05

**Authors:** Theodoros P. Vassilakopoulos, Maria Arapaki, Panagiotis T. Diamantopoulos, Athanasios Liaskas, Fotios Panitsas, Marina P. Siakantaris, Maria Dimou, Styliani I. Kokoris, Sotirios Sachanas, Marina Belia, Chrysovalantou Chatzidimitriou, Elianna A. Konstantinou, John V. Asimakopoulos, Kyriaki Petevi, George Boutsikas, Alexandros Kanellopoulos, Alexia Piperidou, Maria-Ekaterini Lefaki, Angeliki Georgopoulou, Anastasia Kopsaftopoulou, Kalliopi Zerzi, Ioannis Drandakis, Maria N. Dimopoulou, Marie-Christine Kyrtsonis, Panayiotis Tsaftaridis, Eleni Plata, Eleni Variamis, Gerassimos Tsourouflis, Flora N. Kontopidou, Kostas Konstantopoulos, Gerassimos A. Pangalis, Panayiotis Panayiotidis, Maria K. Angelopoulou

**Affiliations:** 1 Department of Haematology and Bone Marrow Transplantation, National and Kapodistrian University of Athens, School of Medicine, Laikon General Hospital, 17 Ag. Thoma Str., 11527 Athens, Greece; arapaki_m@hotmail.gr (M.A.); msdimou@gmail.com (M.D.); beliamarina@hotmail.com (M.B.); cchatzidimitriou@hotmail.com (C.C.); eliankon@hotmail.com (E.A.K.); jv.asimakopoulos@gmail.com (J.V.A.); akanell@hotmail.com (A.K.); pantsaftar@gmail.com (P.T.); mkangelop@gmail.com (M.K.A.); 2 First Department of Internal Medicine, National and Kapodistrian University of Athens, School of Medicine, Laikon General Hospital, 17 Ag. Thoma Str., 11527 Athens, Greece; pandiamantopoulos@gmail.com (P.T.D.);; 3 First Department of Internal Medicine Propedeutic, National and Kapodistrian University of Athens, School of Medicine, Laikon General Hospital, 17 Ag. Thoma Str., 11527 Athens, Greece; 4 Second Department of Surgery Propedeutic, National and Kapodistrian University of Athens, Laikon General Hospital, 11527Athens, Greece; 5 Second Department of Internal Medicine, National and Kapodistrian University of Athens, Ippokration General Hospital, 11527 Athens, Greece; florankontopidou@hotmail.com

**Keywords:** Hodgkin’s lymphoma, β_2_-microglobulin, prognostic factors, chemotherapy

## Abstract

**Simple Summary:**

The significance of serum beta-2 microglobulin (sβ_2_m) in Hodgkin lymphoma (HL) is controversial. In an effort to investigate the prognostic significance of sβ_2_m levels in a large series of patients with HL, we analyzed 915 patients, who were treated with ABVD or equivalent regimens with or without radiotherapy. Sβ_2_m levels were measured by a radioimmunoassay (upper normal limit 2.4 mg/L). The median sβ_2_m levels were 2.20 mg/L. Freedom from progression (FFP) was significantly inferior in patients with a higher sβ_2_m at all tested cutoffs. The best cutoff was 2.0 mg/L (10-year FFP 83% vs. 70%, *p* = 0.001), which performed better than the 2.4 mg/L cutoff (“normal versus high”). Our data suggest that higher sβ_2_m is a significant independent predictor of FFP, OS and HLSS in HL but the optimal cutoff appears to lie within the normal limits in this predominantly young patient population.

**Abstract:**

The significance of serum beta-2 microglobulin (sβ_2_m) in Hodgkin lymphoma (HL) is controversial. We analyzed 915 patients with HL, who were treated with ABVD or equivalent regimens with or without radiotherapy. Sβ_2_m levels were measured by a radioimmunoassay (upper normal limit 2.4 mg/L). Sequential cutoffs (1.8–3.0 by 0.1 mg/L increments, 3.5 and 4.0 mg/L) were tested along with ROC analysis. The median sβ_2_m levels were 2.20 mg/L and were elevated (>2.4 mg/L) in 383/915 patients (41.9%). Higher sβ_2_m was associated with inferior freedom from progression (FFP) at all tested cutoffs. The best cutoff was 2.0 mg/L (10-year FFP 83% vs. 70%, *p* = 0.001), which performed better than the 2.4 mg/L cutoff (“normal versus high”). In multivariate analysis, sβ_2_m > 2.0 mg/L was an independent adverse prognostic factor in the whole patient population. In multivariate overall survival analysis, sβ_2_m levels were predictive at 2.0 mg/L cutoff in the whole patient population and in advanced stages. Similarly, sβ_2_m > 2.0 mg/L independently predicted inferior HL-specific survival in the whole patient population. Our data suggest that higher sβ_2_m is an independent predictor of outcome in HL but the optimal cutoff lies within the normal limits (i.e., at 2.0 mg/L) in this predominantly young patient population, performing much better than a “normal versus high” cutoff set at 2.4 mg/L.

## 1. Introduction

The prognosis of Hodgkin lymphoma (HL) has dramatically changed over the last few decades, with the 5-year survival rate below 10% in the 1960s increasing to a 10-year survival rate exceeding 80% in the 2010s [1,2]. A further increase is expected with the use of novel immunotherapies for relapsed/refractory disease [3,4,5,6] or even their incorporation in earlier treatment lines [7,8]. The prognosis primarily depends on clinical stage as defined by the anatomic extent of the disease and the presence of B-symptoms according to the Ann Arbor staging system [9] and the Cotswolds [10] and Lugano modifications [11]. In early-stage disease, the presence of bulky mediastinal disease, the number of involved nodal sites, elevated erythrocyte sedimentation rate (ESR), extranodal involvement and age provide additional prognostic information [12,13,14,15,16,17,18,19,20,21,22,23,24], while the 7-factor international prognostic score (IPS) has become the standard prognostic tool for advanced stages [25] followed by simplified versions [26,27]. Another 7-factor advanced-stage Hodgkin lymphoma International Prognostic Index was recently published by the HoLISTIC consortium including significantly overlapping factors compared to IPS, albeit handled in a totally different logistic way [28].

Unfortunately, the above and other conventional prognostic systems cannot accurately classify patients with highly divergent levels of risk of relapse/progression and are unable to define either a very low-risk subgroup or any sizeable subgroup of patients with a>40-50% failure rate under modern ABVD-like fixed or Positron Emission Tomography driven therapy [25,26,27,28,29,30]. Many biological prognostic factors have been evaluated in this context, but none has been adopted in everyday prognostication for several reasons [30]. Thus, research is still focusing to the identification of novel, powerful, conventional and biological prognostic factors which might permit the reduction in chemotherapy and omission radiotherapy (RT) in an effort to minimize the long-term toxic effects in low-risk patients and guide the intensification/modification of treatment in high-risk groups.

Serum beta_2_-microglobulin (sβ_2_m) is a well-established prognostic factor in multiple myeloma, and has been incorporated in the international staging system (ISS) [31]. It is also an extensively evaluated prognostic factor in diffuse large B-cell lymphoma and other non-Hodgkin lymphoma subtypes [32,33,34,35,36,37,38,39,40,41,42,43,44,45,46] and may work in acute myeloid leukemia as well [47], but has not been incorporated in current prognostic models for these diseases. Although tested as a prognostic factor in HL 30 years ago [48], its role has not yet been fully established; thus, sβ_2_m has not been used in any of the current prognostic systems for HL. However, there are several small- to medium-sized studies evaluating the potential prognostic role of sβ2m levels in patients with HL using heterogenous therapy, different endpoints, and various cutoffs with conflicting results [48,49,50,51,52,53,54,55,56]. The data from our group, published in 2002 and 2005 based on patients treated with ABVD or equivalent regimens, were also conflicting [52,53]. The updated analysis of 379 patients in 2005 demonstrated a role of sβ_2_m in predicting overall survival and also in predicting failure-free survival in the early stages only [53].

At this point, we extended our series to include 915 patients treated optimally with ABVD or equivalent regimens with or without RT with a much longer median follow-up of approximately 9 years extending up to almost 30 years. The size of this population is by far the largest ever recruited and permits the extraction of much more reliable conclusions. It also enables the reliable evaluation of multiple cutoffs, since the prognostic significance of sβ_2_m levels is not necessarily evident in a “normal versus elevated” analysis and optimal cutoffs for clinical use may also be different in different disease stages.

## 2. Patients and Methods

### 2.1. Patients, Staging, Treatment Strategies and Laboratory Assays

We analyzed 915 patients who received a diagnosis and first-line treatment for HL between 1990 and 2018, and had available sβ_2_m levels at diagnosis. The study period was extended from the beginning of sβ_2_m-level determination in clinical practice until the change in the method of sβ_2_m measurement implying a different cutoff in 2018. All patients were older than 14 years, were HIV-negative, and had received treatment with anthracycline-based CT with or without RT. In this retrospective study, patients were selected solely based on the availability of pretreatment sβ_2_m levels and their characteristics were comparable with those of patients who had also received anthracycline-based chemotherapy with or without RT during the same period, but did not have available serum β_2_-microglobulin levels, as previously reported [52,53].

All patients were clinically staged according to the Ann Arbor system [9], using standard staging procedures. Clinical Ann Arbor stages (AAS) IA and IIA were considered early, while clinical stages IB, IIB, III and IV were considered advanced for the purposes of this analysis. The number of involved anatomic sites was determined as described in previous publication of our group. Hemoglobin, white blood cell counts, and the differential erythrocyte sedimentation rate (ESR), serum albumin and serum LDH levels were measured by standard assays. Anemia was defined as the presence of hemoglobin levels <13 g/dL for males and <11.5 g/dL for females. Serum albumin was analyzed at a cutoff of 4 g/dL, as proposed by the IPS [25]. Severe lymphopenia was also defined according to the cutoff provided by the IPS (<0.6 × 10^9^/L or <8%) [25].

Treatment strategies for early (IA, IIA) and advanced AAS (IB, IIB, III, IV) patients have been described previously [52,57]. PET-driven strategies have been adopted during the last 15 years, initially for advanced- and later for early-stage disease. The evolution of treatment strategies during the study period has been described in recent publications of our group [58,59].

Sβ2m was measured using a radioimmunoassay (Pharmacia). The range of normal values was 1.0–2.4 mg/L.

The study was approved by the appropriate Institutional Review Board. As a non-interventional retrospective study, informed consent was waived.

### 2.2. Statistical Analysis

The frequency of elevated sβ_2_m levels among various subgroups of patients were compared by the chi-square test. The Mann–Whitney and Kruskal–Wallis tests were used for non-parametric comparisons, as appropriate. The correlation between sβ_2_m levels and other variables evaluated as continuous was estimated by the Spearman’s rho coefficient. The optimal cutoff for sβ_2_m levels was determined by direct testing of sequential cutoffs and by the use of Receiver Operator Curves (ROCs). The results obtained by both approaches were very similar.

Freedom from progression (FFP) was defined as the time interval between treatment initiation and treatment failure or last follow-up. Treatment failure was defined as the inability to achieve complete or partial remission (CR, PR) during initial therapy, requiring a switch to alternative chemotherapy, or relapse/progression after an initial CR/PR or toxic death. Patients with deaths of unrelated causes were censored. Overall survival (OS) and Hodgkin lymphoma-specific survival (HLSS) were measured from treatment initiation to death from any cause or HL-related causes (progressive HL, death of treatment toxicity), respectively, or last follow-up. Deaths due to secondary malignancies or cardiovascular causes during CR were censored. Survival after failure (SAF) was defined as the time interval between the documentation of treatment failure (primary failure or relapse) and death from any cause or last follow-up. The estimation of actuarial FFP or survival was performed using the Kaplan–Meier method [60]. The identification of prognostic factors in univariate analysis was based on the log-rank test [61]. The identification of independent prognostic factors was performed using Cox’s proportional hazards model [62].

## 3. Results

### 3.1. Patients’ Characteristics

The median age of the patients was 32 years (14–86) and 513 (56.1%) were males. Among 915 patients, 515 (56.3%) had early- and 400 (33.7%) had advanced-stage disease, while 304 (33.2%) had B-symptoms. The histologic subtype of 891 patients with recorded information was nodular sclerosis in 610 (68.5%), mixed cellularity in 173 (19.4%), nodular lymphocyte predominance in 44 (4.9%), lymphocyte rich classical in 39 (4.4%), lymphocyte depletion in 3 (0.3%), and classical HL unclassified, overlapping or interfollicular in 22 (2.4%). In general, patients’ characteristics were compatible with other reported unselected series of patients with non-pediatric HL. As patients had been diagnosed between 1990 and 2018, the median follow-up of those who were alive at the time of the analysis, was 105.1 months (1.6–353.7).

### 3.2. Serum β_2_-Microglobulin Levels and Clinicopathologic Correlations

The median observed sβ_2_m levels were 2.20 mg/L, with an interquartile range (IQR) of 1.80-3.00 mg/L and a range of 0.50-14.40 mg/L. Elevated sβ_2_m levels (>2.40 mg/L) were found in 383/915 patients (41.9%).

The correlation between sβ_2_m levels and other potential prognostic factors is shown in Table 1. Sβ_2_m levels correlated strongly with all baseline features, including demographics (older age, male gender), non-nodular sclerosing classical HL, clinical and laboratory markers of disease extent and aggressiveness and the IPS (all *p*-values <0.001), with only correlations with leukocytosis, iliac/inguinal and lung involvement being looser but still statistically significant.

With respect to potential biological prognostic factors, highly significant correlations of moderate magnitude were observed between sβ_2_m and serum soluble CD30, serum interleukin-10 and serum ferritin (*p* < 0.001 but Spearman’s rho 0.333–0.455), as summarized in Table 2. However, there were no significant associations with bcl-2, activated caspase-3 or Epstein–Barr virus Latent Protein-1 (LMP-1) immunohistochemical expression.

### 3.3. Freedom from Progression

The 10-year FFP rate for the whole series was 76%. Among 208 events, only 3 were toxic deaths, while 205 were related to progressive or relapsing disease. As expected, most of the potential prognostic factors listed in Table 1 were statistically significant in the univariate analysis of FFP at the level of ≤0.001, with the exception of iliac/inguinal involvement (*p* = 0.047), age, gender, histology, leukocytosis, lung involvement and the number of nodal sites (in advanced disease only), which were not significant.

#### 3.3.1. All Patients

**Figure 1:**
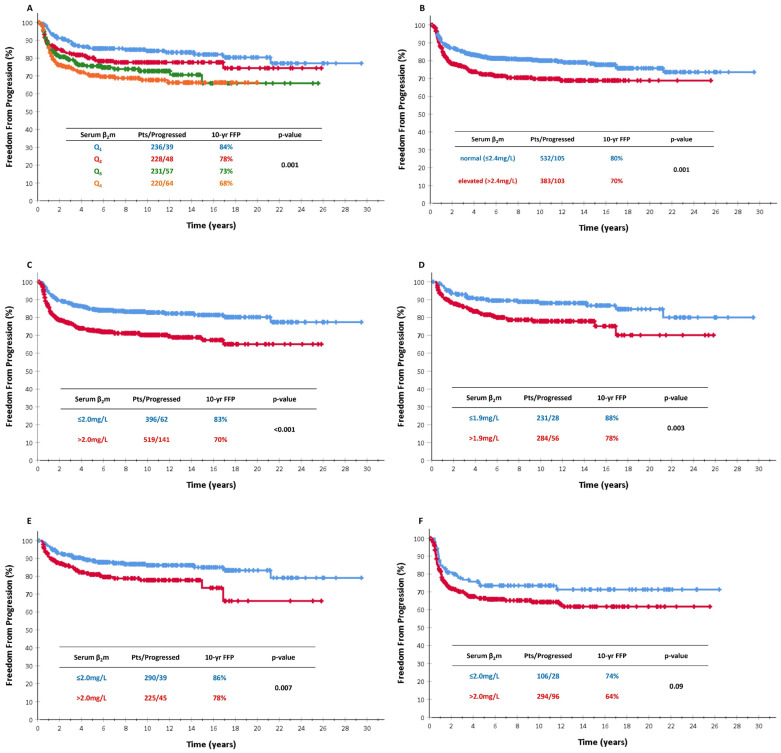
(**A**) Freedom from progression (FFP) according to sβ_2_m levels classified as quartiles in the whole patient population; (**B**) FFP according to sβ_2_m levels as “normal vs. elevated” in the whole patient population (≤2.4 mg/L vs. >2.4 mg/L); (**C**) FFP according to sβ_2_m levels (≤2.0 mg/L vs. >2.0 mg/L) in the whole patient population; (**D**) FFP according to sβ_2_m levels (≤1.9 mg/L vs. >1.9 mg/L) in early-stage patients; (**E**) FFP according to sβ_2_m levels (≤2.0 mg/L vs. >2.0 mg/L) in early-stage patients; and (**F**) FFP according to sβ_2_m levels (≤2.0 mg/L vs. >2.0 mg/L)in advanced-stage patients.

When sβ_2_m levels were classified as quartiles, a consistent drop of 5–6% was observed for each one from Q1 to Q4, with 10-year rates of 84%, 78%, 73% and 68% (*p* = 0.001, Figure 1A), indicating a “dose–response” effect.

Patients with elevated sβ_2_m levels had inferior FFP (70% versus 80%, *p* = 0.001, Figure 1B). As the “normal versus elevated” comparison is arbitrary and may not be optimal, several cutoff points for sβ_2_m levels were evaluated to identify the optimal cutoff to predict FFP, starting from 1.8 mg/L and advancing in 0.1 mg/L steps up to 3.0 mg/L and then at 0.5 mg/L steps up to 4.0 mg/L. In the univariate analysis, FFP was significantly inferior in patients with higher sβ2m at all tested cutoffs, as shown in Table 3. Interestingly, the 2.4 mg/L cutoff (“normal versus elevated”) was not the best one, as the widest difference was observed at the cutoff of 2.0 mg/L(10-year FFP 83% versus 70%, *p* < 0.001; Figure 1C). ROC curve analysis confirmed this finding and provided a best cutoff at 2.02 mg/L. The area under the curve (AUC) was 0.573 (95% CI 0.53–0.62; *p* = 0.01).

sβ_2_m levels >2.0 mg/L were an independent adverse prognostic factor in the large-scale multivariate analysis (see Table 4 footnote) of all 915 patients, along with stage and lymphocytopenia (hazard ratio (HR) 1.55, 95% confidence intervals (CI) 1.11–2.17, *p* = 0.01; Table 4). The “normal versus elevated” comparison was not significant in the multivariate analysis (Table 4).

#### 3.3.2. Early Stages

Among 515 patients with early-stage HL (IA/IIA), the best cutoff was found at 1.9 mg/L, with 10-year FFP rates of 88% versus 78% (*p* = 0.003, Figure 1D). Significant results were also obtained at the cutoff of 2.0 mg/L, with 10-year FFP rates of 86% versus 78% (*p* = 0.007, Figure 1E). As shown in Table 3, cutoffs set at 2.2 mg/L or higher, including the “normal versus elevated” comparison were not predictive of FFP. Sβ_2_m levels > 2.0 mg/L were an independent adverse prognostic factor in a large-scale multivariate analysis of patients with early stages along with ≥3 nodal sites and ESR ≥ 50 mm/h (hazard ratio (HR) 1.65, 95% confidence intervals (CI) 1.04–2.62, and *p* = 0.034; Table 4). Unexpectedly, the “normal versus elevated” comparison was also significant in multivariate analysis with a similar HR (Table 4).

#### 3.3.3. Advanced Stages

Among 400 patients with advanced-stage HL (IB/IIB/III/IV), none of the tested cutoffs, including the “normal versus elevated” comparison, were predictive of FFP in univariate analysis (Table 3). The best cutoff was set at 2.0 mg/Land resulted in a marginally significant prediction, with 10-year FFP rates of 74% versus 64% (*p* = 0.09, Figure 1F). Similarly to the univariate results, sβ_2_m levels > 2.0 mg/L were an independent adverse prognostic factor of borderline significance in the multivariate analysis of patients with advanced stages, including all the IPS factors (Table 4; see also footnote) along with stage IV, lymphopenia and leukocytosis (protective!!) (hazard ratio (HR) 1.44, 95% CI 0.94–2.21, and *p* = 0.098; Table 4). The “normal versus elevated” comparison was not significant in multivariate analysis (Table 4).

### 3.4. Overall Survival

The 10-year OS rate for the whole series was 85%. Among 131 deaths, 74 were disease-related and 57 unrelated.

#### 3.4.1. All Patients

**Figure 2:**
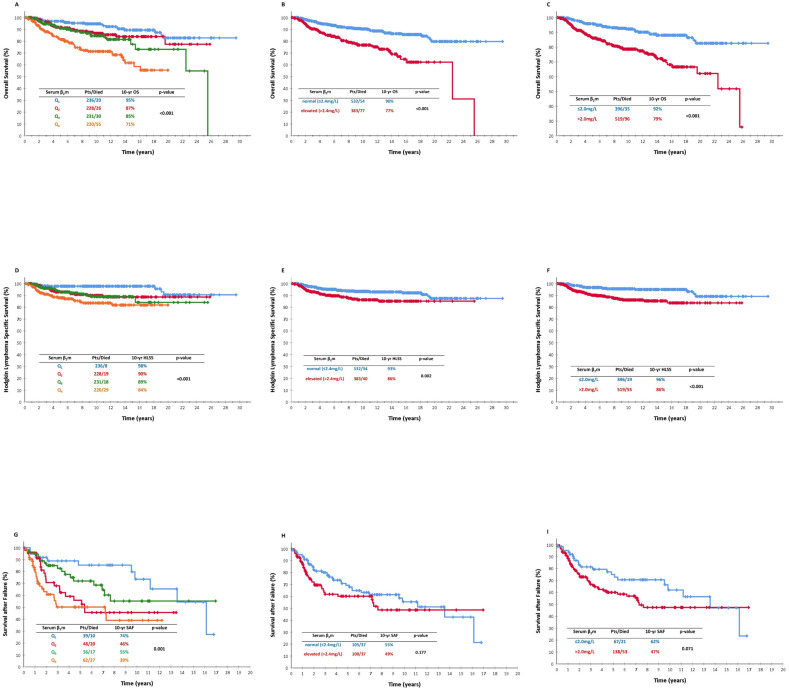
(**A**) Overall survival (OS) according to sβ_2_m levels classified as quartiles in the whole patient population; (**B**) OS according to sβ_2_m levels as “normal vs. elevated” in the whole patient population (≤2.4 mg/L vs. >2.4 mg/L); (**C**) OS according to sβ_2_m levels (≤2.0 mg/L vs. >2.0 mg/L) in the whole patient population; (**D**) Hodgkin lymphoma-specific survival (HLSS) according to sβ_2_m levels classified as quartiles in the whole patient population; (**E**) HLSS according to sβ_2_m levels as “normal vs. elevated” in the whole patient population (≤2.4 mg/L vs. >2.4 mg/L); (**F**) HLSS according to sβ_2_m levels (≤2.0 mg/L vs. >2.0 mg/L) in the whole patient population; (**G**) survival after failure (SAF) according to sβ_2_m levels classified as quartiles in the whole patient population; (**H**) SAF according to sβ_2_m levels as “normal vs. elevated” in the whole patient population (≤2.4 mg/L vs. >2.4 mg/L); and (**I**) SAF according to sβ_2_m levels (≤2.0 mg/L vs. 2.0 mg/L) in the whole patient population.

When sβ_2_m levels were classified as quartiles, a gradual drop was observed for each one from Q1 to Q4, with 10-year rates of 95%, 87%, 85% and 71% (*p* < 0.001; Figure 2A).

Patients with elevated sβ_2_m levels had inferior OS (90% versus 77%, *p* < 0.001; Figure 2B). At the cutoff of 2.0 mg/L, the difference was similar (92% versus 79%, *p* < 0.001; Figure 2C). Sβ2m levels > 2.0 mg/L were an independent adverse prognostic factor in a large-scale multivariate analysis of all 915 patients (see Table 2 footnote), along with older age, B-symptoms and lymphocytopenia (hazard ratio (HR) 1.96, 95% CI 1.21–3.19, and *p* = 0.006; Table 4). The “normal versus elevated” comparison yielded borderline results in the multivariate analysis (Table 4).

#### 3.4.2. Early and Advanced Stages

In the multivariate analysis of OS in early-stage patients, sβ_2_m levels were neither an independent prognostic factor at the cutoff of 2.0 mg/L nor at a “normal versus elevated” basis (Table 4). In contrast, in advanced stages, sβ_2_m > 2.0 mg/L was an independent adverse prognostic factor along with older age, lymphopenia, anemia and a lack of leukocytosis (hazard ratio (HR) 2.07, 95% CI1.04-4.15, and *p* = 0.039; Table 4). The “normal versus elevated” comparison was not predictive in advanced-stage disease (Table 4).

### 3.5. Causes of Death, Hodgkin Lymphoma-Specific Survival and Survival after Failure

Up to publication of this study, 131 deaths were recorded. Among them, 74 (56%) were due to HL, with 58 being directly related to progressive HL, 5 toxic deaths (3 during first-line and 2 during salvage therapy), 9 secondary neoplasias plus active HL and 1 congestive heart failure directly after treatment. We also recorded 27 unrelated deaths and 30 deaths of secondary neoplasia during first complete remission.

The 10-year HLSS rate for the whole series was 91%. When sβ_2_m levels were classified as quartiles, a gradual drop was observed for each one from Q1 to Q4, with 10-year rates of 98%, 90%, 89% and 84% (*p* < 0.001; Figure 2D).

Patients with elevated sβ_2_m levels had inferior HLSS (93% versus 86%, *p* = 0.002; Figure 2E). At the cutoff of 2.0 mg/L, the difference was even more marked (96% versus 86%, *p* < 0.001; Figure 2F). Sβ2m levels > 2.0 mg/L were an independent adverse prognostic factor in the large-scale multivariate analysis (see Table 4 footnote) of all 915 patients, along with B-symptoms, lymphopenia and a lack of leukocytosis (hazard ratio (HR) 2.21, 95% CI 1.19–4.11, and *p* = 0.012; Table 4). The “normal versus elevated” comparison did not lead to a statistically significant result in the multivariate analysis (Table 4).

In the multivariate analysis of HLSS in early-stage patients, sβ_2_m levels were the only variable with a borderline-independent effect, only when evaluated at the cutoff of 2.0 mg/L (hazard ratio (HR) 2.30, 95% CI 0.89–5.94, and *p* = 0.085; Table 4), but not in a “normal versus elevated” basis (Table 4). In contrast, in the multivariate analysis of HLSS in advanced stages, sβ_2_m levels were neither an independent prognostic factor at the cutoff of 2.0 mg/L nor at a “normal versus elevated” basis (Table 4).

The 10-year SAF rate for the whole series was 61%. A statistically significant impact on SAF was observed when levels were classified as quartiles, with 10-year rates of 74%, 46%, 55% and 39% for Q1 through Q4 (*p* = 0.001; Figure 2G). Patients with elevated sβ_2_m levels had a similar SAF to those with normal levels (49% versus 55%, respectively, *p* = 0.177; Figure 2H). At the cutoff of 2.0 mg/L, the difference became borderline (62% versus 47%, *p* = 0.071; Figure 2I).

## 4. Discussion

B_2_m is synthesized in all nucleated cells, binds to major histocompatibility complex class I molecules, and is not directly attached to the cell membrane. Thus, free soluble β_2_m is detected in body fluids due to its release from the cell surface and cytoplasm. Since its identification 50 years ago, in 1972 [63], β_2_m has been widely investigated as a prognostic factor in hematologic malignancies. Further to the correlation with tumor burden [52], the mechanisms underlying the prognostic significance of β_2_mstill remain unclear. Indeed, several studies have shown that the prognostic significance of sβ_2_mmay be independent from factors reflecting disease burden [33,35], suggesting that it could either be related to other specific biologic features of lymphomas or simply overcome other markers of tumor burden, obscuring their significance.

The role of sβ_2_m as a prognostic factor in HL has been evaluated in several small- or medium-sized studies in the past, with partially conflicting results, probably owing to the sample sizes and the variable treatment approaches (Table 5) ([48,49,50,51,53,55,56,64,65,66,67,68,69], present study). Briefly, considering both the MD Anderson studies together [48,51], sβ_2_m appears to correlate with FFS in advanced stages (overall survival not reported), while it was associated with inferior overall survival in early stages, with only a borderline effect on FFS. It should be noted that treatment was inferior to ABVD and equivalents in the majority of these patients. In a study of the International Hodgkin Study Group, sβ_2_m was an independent predictor of FFS in early stage patients with favorable characteristics treated with RT alone [66].

In the present study, we evaluated the prognostic role of sβ_2_m in HL in the—by far—largest series published to date, consisting of a large cohort of homogeneously treated patients. Importantly, all patients had been treated with ABVD or equivalent regimens with or without RT, which are considered standard therapy for HL. Since it is known that more effective treatment may eliminate the significance of previously established prognostic factors, our study rules out a potential bias due to inferior treatment. Our results extend our previous observations and establish sβ_2_m as a potential independent prognostic factor in HL.

An important novel observation was made possible thanks to the very large size of this patient population: analyzing several potential cutoffs, we concluded that sβ_2_m may not work well as a prognostic factor, neither when analyzed on a “normal versus elevated” basis at the cutoff of 2.4 mg/L(as performed in our previous studies) [52,53] nor when analyzed at higher—clearly abnormal—cutoffs, as 2.5 mg/L [48,51,55,56] or 3.0 mg/L [65]. Instead, sβ_2_m worked better when the cutoff was set within the normal range at 2.0 mg/L. It is reasonable to wonder whether this observation is biologically relevant. In our opinion it is reasonable, because sβ_2_m levels are strongly and positively correlated with age in normal subjects. As the normal range is established from unselected normal individuals from the general population, the true upper normal limit for younger people might probably be lower. Along these lines, as patients with HL are much younger than the general population, the expected upper normal limit of sβ_2_m for the majority of them might be lower than the conventional 2.4 mg/L and might approach 2.0 mg/L.

The use of a sβ_2_m cutoff within the normal range of is supported by a recent Chinese study, in which 353 patients were evaluated, among whom 230 had received ABVD and 123 ABVD-like regimens, the latter with inferior progression-free survival (PFS). The levels of sβ_2_m were evaluated by ROC curves and the best cutoff was set at 1.85 mg/L, very similarly to our results. Although sβ_2_m levels above that cutoff were associated with inferior PFS and OS in the univariate analysis, the prognostic significance was independent of other factors only for OS. This is not unexpected, as sβ_2_m levels are more potent predictors of OS, as shown in the present and our previous studies, because of their strong association with age and renal function. However, the moderate size of the study by Wen et al. might have obscured an independent effect of sβ_2_m levels on PFS. In another small study of 67 patients, ROC analysis suggested a cutoff of 2.5 mg/L, which produced significant results in multivariate analysis for PFS, OS and DSS [55].

Reporting here our experience in 915 patients with HL, with 208 treatment failure events recorded so far (84 in early and 124 in advanced stages), this study was powered to detect moderate but clinically significant differences and to perform subgroup analyses according to clinical stage. Serum β_2_mlevels >2.0 mg/L independently predicted a lower FFP rate in the whole-patient population of this study, when evaluated in multivariate analysis including 11 additional and potentially strong prognostic covariates. The same was true for OS and HLSS. Notably, on a “normal versus elevated” basis, sβ_2_m had no independent effect on FFP and HLSS, presenting only a borderline association with OS. Among 515 patients with early-stage HL (IA/IIA), the best cutoff was found at 1.9 mg/L, but 2.0 mg/L was also highly significant and was used for further evaluation for reasons of consistency. Again, sβ_2_m was an independent predictor of FFP, when evaluated in multivariate analysis including eight additional covariates with established or strongly suspected prognostic significance in early-stage disease. Serum β_2_m levels were the only independent predictor of HLSS, albeit of marginal significance, but had no effect on OS. Among 400 patients with advanced-stage HL (IB/IIB/III/IV), sβ_2_m levels >2.0 mg/L were an independent predictor of FFP, when evaluated in multivariate analysis including all seven IPS covariates. This effect was more marked regarding OS but sβ_2_m had no independent effect on HLSS. On a “normal versus elevated” basis, sβ_2_m had no independent effect in any of the three endpoints.

Finally, this is the first study to show that sβ_2_m levels are significantly correlated with some established biological prognostic factors, such as serum sCD30 and serum IL-10 levels [66], but not with others, such as the tissue expression of bcl-2 and activated caspase-3 [30].

## 5. Conclusions

The present study has established the prognostic impact of sβ_2_m at a lower-than-expected cutoff, but also raises several new questions. It is not clear if sβ_2_mcan add to the prediction achieved by the IPS or other prognostics systems or just replace variables within the existing systems. Unfortunately, sβ_2_m was not evaluated during the development of the new holistic IPS [28], while its additive impact when biological prognostic factors are taken into account remains unknown. Some kind of “correction” according to the renal function should also be investigated in the effort to increase the prognostic significance of sβ_2_m levels. Another problem is that interim PET-guided therapy has predominated the field of treatment of Hodgkin lymphoma during the last decade. It is not clear whether sβ_2_m simply predicts a higher probability of interim PET positivity or may further help to discriminate which patients with a negative interim PET will relapse or who will be cured with intensified treatment following a positive interim PET. Similar considerations apply regarding the potential association of sβ_2_m levels with the results of end-of-treatment PET [70]. In addition, as novel prognostic factors appear, their correlation with sβ_2_m should be accurately determined. Unfortunately, there are no data regarding the correlation of sβ_2_m, neither with the circulating tumor DNA [71,72,73] and its changes during treatment nor with PET metrics, including baseline total metabolic tumor volume (TMTV) [74,75,76,77,78], total lesion glucolysis (TLG) [79,80] or lesion dissemination [81,82,83]. As sβ_2_m levels correlated strongly with almost all baseline features reflecting disease extent and aggressiveness in this study, it is reasonable to hypothesize a strong correlation with PET metrics, which, however, does not exclude the persistence of the independent prognostic significance of sβ_2_m.Finally, probably the main question to be asked in the near future is how sβ_2_m will affect the outcome of patients treated in the first line with chemotherapy plus novel agents such as BV-AVD or BreCADD, or—more importantly—how sβ_2_m will work as a prognostic factor under treatment with nivolumab-AVD [8].

## Figures and Tables

**Table 1:** Patient characteristics and correlations with serum β_2_-microglobulin levels.

Patient Characteristics	Value	Patients		Serum β_2_-Microglobulin		*p*-Value
		#	%	Median	IQR	
Age (years)	<45 ≥45	659 256	72.0 28.0	2.00 3.00	1.70–2.63 2.20–4.20	<0.001
Gender	female male	402 513	43.9 56.1	2.00 2.37	1.70–2.80 1.89–3.20	<0.001
AnnArbor Stage	I/IIA IB/IIB/III/IV	515 400	56.3 33.7	1.98 2.70	1.70–2.55 2.00–3.70	<0.001
AnnArbor Stage	IA/B IIA/B IIIA/B IVA/B	182/13 320/111 75/87 34/93	19.9/1.4 35.0/12.1 8.2/9.5 3.7/10.2	2.00/2.90 1.95/2.30 2.40/3.20 2.49/2.93	0.85/2.15 0.82/1.54 1.40/1.96 1.59/2.03	<0.001
B-Symptoms	A B	611 304	66.8 33.2	2.00 2.80	1.70–2.61 2.03–3.93	<0.001
Histology	NLP NS MC LD LR UCL IF-NS/MC	44 610 173 3 39 16 4+2	4.9 68.5 19.4 0.3 4.4 1.8 0.6	1.90 2.13 2.61 2.48 2.30 3.39 1.90	1.51–2.58 1.76–2.80 1.90–3.70 1.90–5.30 1.90–2.95 2.38–4.33 1.87–3.04	<0.001
Bone marrow involvement	no yes	819 41	95.2 4.8	2.17 3.50	1.78–3.00 2.68–5.25	<0.001
Liver involvement	no yes	887 21	97.7 2.3	2.20 4.00	1.80–3.00 2.74–4.76	<0.001
Lung involvement	no yes	819 84	90.7 9.3	2.18 2.44	1.78–3.00 2.00–3.48	0.012
Iliac/inguinal involvement	no yes	818 82	90.9 9.1	2.10 3.00	1.77–2.80 2.03–3.80	0.022
Anemia	no yes	525 389	57.4 42.6	2.00 2.52	1.70–2.63 1.95–3.70	<0.001
Leukocytosis (×10^9^/L)	<10 ≥10	533 377	58.6 41.4	2.20 2.22	1.79–3.24 1.82–2.90	0.83
Marked Leukocytosis(×10^9^/L)	<15 ≥15	787 123	86.5 13.5	2.18 2.40	1.78–3.00 1.97–3.14	0.019
Severe Lymphocytopenia	no yes	771 97	88.8 11.2	2.20 2.54	1.80–3.00 2.02–3.84	<0.001
ESR (mm/h)	<50 ≥50	408 411	49.8 50.2	2.00 2.41	1.70–2.60 1.90–3.38	<0.001
LDH	normal elevated	599 251	70.5 29.5	2.10 2.61	1.75–2.80 2.00–3.62	<0.001
Albumin (g/dL)	≥4 <4	469 413	53.2 46.8	2.00 2.58	1.70–2.60 1.94–3.68	<0.001
IPS	0–2 3–7	633 244	72.2 27.8	2.00 3.05	1.70–2.60 2.26–4.08	<0.001
Nodal sites (#; AAS I/IIA)	1–2 ≥3	371 143	72.2 27.8	2.00 1.93	1.70–2.60 1.70–2.47	0.69
Involved sites (#; AAS IIB-IV)	≤4 ≥5	205 183	52.8 47.2	2.40 3.00	1.87–3.45 2.21–4.00	<0.001

IQR = interquartile range, NLP = nodular lymphocyte predominant, NS = nodular sclerosis, MC = mixed cellularity, LD = lymphocyte depleted, LR = lymphocyte rich classical, UCL = unclassified classical Hodgkin lymphoma, IF = interfollicular classicl hodgkin lymphoma, NS/MC = classical Hodgkin lymphoma with overlapping features between nodular sclerosis and mixed cellularity, ESR = erythrocyte sedimentation rate, LDH = serum lactate dehydrogenase, IPS = international prognostic score, # = number, AAS = Ann-Arbor stage.

**Table 2:** Correlation between serum β_2_-microglobulin levels and other—rarely reported—biological prognostic factors.

Biological Prognostic Factor	Patients with Available Data (#)	Statistical Method	*p*-Value	Comments
Serum Ferritin(ng/mL)	399	Spearman’s rho = 0.455	<0.001	Positive correlation
Serum soluble CD30	204	Spearman’s rho = 0.333	<0.001	Positive correlation
Serum interleukin10(pg/mL)	204	Spearman’s rho = 0.336	<0.001	Positive correlation
Bcl-2 expression	102	Mann–Whitney	0.64	-
Activated caspase-3	73	Mann–Whitney	0.79	-
LMP-1 expression	189	Mann–Whitney	0.10	↑β_2_m in positive cases

**Table 3:** Univariate analysis of the prognostic significance of serum β_2_-microglobulin levels on various cutoff points in terms of freedom from progression.

Cutoff	All Patients			Stages IA/IIA			Stages IB/IIB/III/IV		
(mg/L)	Pts/Failed	10y-FFP	*p*	Pts/Failed	10y-FFP	*p*	Pts/Failed	10y-FFP	*p*
≤1.8	236/36	84	0.002	181/22	88	0.012	55/17	70	0.569
>1.8	679/169	73		334/62	79		345/107	66	
≤1.9	311/52	84	<0.001	231/28	88	0.003	80/24	70	0.431
>1.9	604/156	72		284/56	78		320/100	66	
≤2.0	396/67	83	<0.001	290/39	86	0.007	106/28	71	0.090
>2.0	519/141	70		225/45	78		294/96	64	
≤2.1	424/75	82	<0.001	304/41	86	0.007	120/34	71	0.211
>2.1	491/133	70		211/43	77		280/90	65	
≤2.2	464/87	81	<0.001	326/48	85	0.07	138/39	71	0.182
>2.2	451/121	70		189/36	77		262/85	64	
≤2.3	496/97	80	0.001	345/51	85	0.063	151/46	70	0.477
>2.3	419/111	70		170/33	78		249/78	65	
≤2.4	532/105	80	0.001	365/54	85	0.06	167/51	70	0.504
>2.4	393/105	70		150/30	78		233/73	65	
≤2.5	566/113	80	0.001	384/58	84	0.115	182/55	70	0.428
>2.5	349/95	69		131/26	78		218/69	64	
≤2.6	596/117	80	<0.001	401/60	85	0.08	195/57	71	0.243
>2.6	319/91	68		114/29	76		205/67	63	
≤2.7	623/123	80	<0.001	418/65	84	0.286	205/58	72	0.107
>2.7	292/85	67		97/19	78		195/66	62	
≤2.8	646/138	79	0.002	427/69	83	0.743	219/61	70	0.188
>2.8	269/75	69		88/15	81		181/60	63	
≤2.9	664/137	79	0.002	437/70	83	0.600	227/67	70	0.256
>2.9	251/71	68		78/14	80		173/57	60	
≤3.0	695/144	78	0.002	452/73	83	0.714	243/71	70	0.282
>3.0	220/64	68		63/11	80		157/53	63	
≤3.5	759/161	78	0.002	477/77	83	0.538	282/84	69	0.291
>3.5	156/47	67		38/7	81		118/40	63	
≤4.0	810/178	77	0.044	490/80	83	0.887	320/98	68	0.604
>4.0	105/30	68		25/4	82		80/26	64	

FFP = freedom from progression.

**Table 4:** Multivariate analysis of the prognostic significance of serum β_2_-microbulin levels of freedom from progression, and overall and Hodgkin lymphoma-specific survival. Analysis performed at the cutoff of 2.0 mg/L or a “normal vs. elevated” basis (cutoff 2.4 mg/L) in the whole potent population and in early and advanced stages separately.

Covariates Entering the Multivariate Model	Serum β_2_-Microglobulin at the 2.0 mg/L Cutoff			Covariates Entering the Multivariate Model	Serum β_2_-Microglobulin on a “Normal vs. Elevated” Basis		
	Hazard Ratio	95% Cl	*p*-Value		Hazard Ratio	95% Cl	*p*-Value
All patients—Freedom From Progression *							
Clinical Stage				Clinical Stage			
Stage IIB/III vs. I/IIA	1.65	1.16–2.36	0.005	Stage IIB/III vs. I/IIA	1.84	1.30–2.60	0.001
Stage IV vs. I/IIA	2.29	1.53–3.42	<0.001	Stage IV vs. I/IIA	2.59	1.75–3.85	<0.001
Lymphopenia (yes vs. no)	1.76	1.19–2.59	0.004	Lymphopenia (yes vs. no)	1.84	1.24–2.72	0.002
Sβ_2_m (>2.0 vs. ≤2 mg/L)	1.55	1.11–2.17	0.01	Sβ_2_m (>2.4 vs. ≤2.4 mg/L)	Not	selected	-
Early stages—Freedom From Progression (I/IIA) **							
Nodal Sites # (≥3 vs.<3)	1.97	1.24–3.16	0.005	Nodal Sites # (≥3 vs. <3)	2.00	1.24–3.21	0.004
ESR (≥50 vs.<50 mm/h)	1.52	0.94–2.45	0.085	ESR (≥50 vs.<50 mm/h)	1.58	0.98–2.53	0.059
Sβ_2_m(>2.0 vs. ≤2 mg/L)	1.65	1.04–2.62	0.034	Sβ_2_m (>2.4 vs. ≤2.4 mg/L)	1.67	1.03–2.72	0.038
Advanced Stages—Freedom From Progression (IIB/III/IV) ***							
Lymphopenia (yes vs. no)	2.31	1.51–3.54	<0.001	Lymphopenia (yes vs. no)	2.36	1.54–3.61	<0.001
WBC (≥15 vs. <15 × 10^9^/L)	0.61	0.38–0.99	0.047	WBC (≥15 vs. <15 × 10^9^/L)	0.62	0.38–1.02	0.058
Stage (IV vs. IB/IIB/III)	1.42	0.98–2.06	0.067	Stage (IV vs. IB/IIB/III)	1.44	0.99–2.08	0.057
Sβ_2_m (>2.0 vs. ≤2 mg/L)	1.44	0.94–2.21	0.098	Sβ_2_m (>2.4 vs. ≤2.4 mg/L)	not	selected	-
All Patients—Overall Survival *							
Age (≥45 vs. <45 years)	2.63	1.73–3.99	<0.001	Age (≥45 vs.<45)	2.64	1.70–4.07	<0.001
B-symptoms (yes vs. no)	2.01	1.31–3.07	0.001	B–symptoms (yes vs. no)	2.07	1.34–3.18	0.001
Lymphopenia (yes vs. no)	1.83	1.07–3.12	0.027	Lymphopenia (yes vs. no)	1.84	1.08–3.15	0.021
Sβ_2_m (>2.0 vs. ≤2 mg/L)	1.96	1.21–3.19	0.006	Sβ_2_m (>2.4 vs. ≤2.4 mg/L)	1.53	0.97-2.41	0.067
Early Stages (I/IIA)—Overall Survival **							
Age (≥45 vs. <45 years)	2.34	1.23–4.46	0.01	Age (≥45 vs.<45)	2.34	1.23–4.46	0.010
Gender (male vs. female)	2.25	1.14–4.42	0.019	Gender (male vs. female)	2.25	1.14–4.42	0.019
Sβ_2_m (>2.0 vs. ≤2 mg/L)	Not	selected		Sβ_2_m (>2.4 vs. ≤2.4 mg/L)	Not	selected	
Advanced Stages (IB/IIB/III/IV)—Overall Survival ***							
Age (≥45 vs. <45 years)	4.02	2.44–6.62	<0.001	Age (≥45 vs.<45 years)	4.92	3.03–8.00	<0.001
Lymphopenia (yes vs. no)	2.57	1.46–4.52	0.001	Lymphopenia (yes vs. no)	2.35	1.35–4.08	0.003
Anemia (yes vs. no)	1.74	0.99–3.06	0.054	Anemia (yes vs. no)	1.79	1.03–3.13	0.04
WBC (≥15 vs. <15 × 10^9^/L)	0.58	0.31–1.13	0.10	WBC (≥15 vs. <15 × 10^9^/L)	not	selected	-
Sβ_2_m (>2.0 vs. ≤2 mg/L)	2.07	1.04–4.15	0.039	Sβ_2_m (>2.4 vs. ≤2.4 mg/L)	not	selected	-
All Patients—Hodgkin Lymphoma Specific Survival *							
B-symptoms (yes vs. no)	3.11	1.75–5.53	<0.001	Clinical Stage			
Lymphopenia (yes vs. no)	2.17	1.15–4.09	0.017	Stage IIB/III vs. I/IIA	2.64	1.35–5.18	0.005
WBC (≥10 vs. <10 × 10^9^/L)	0.52	0.30–0.90	0.019	Stage IV vs. I/IIA	3.10	1.43–6.72	0.004
Sβ_2_m (>2.0 vs. ≤2 mg/L)	2.21	1.19–4.11	0.012	Lymphopenia (yes vs. no)	2.76	1.48–5.15	0.001
				WBC (≥10 vs. <10 × 10^9^/L)	0.51	0.29–0.89	0.019
				Anemia (yes vs. no)	1.79	0.97–3.29	0.061
				Sβ_2_m (>2.4 vs. ≤2.4 mg/L)	not	selected	
Early Stages (I/IIA)—Hodgkin Lymphoma Specific Survival **							
Sβ_2_m (>2.0 vs. ≤2 mg/L)	2.30	0.89–5.94	0.085	No model fitted			
Advanced Stages (IB/IIB/III/IV)—Hodgkin Lymphoma Specific Survival ***							
Age (≥45 vs. <45 years)	2.45	1.38–4.36	0.002	Age (≥45 vs.<45 years)	2.45	1.38–4.36	0.002
Lymphopenia (yes vs. no)	2.99	1.57–5.68	0.001	Lymphopenia (yes vs. no)	2.99	1.57–5.68	0.001
Anemia (yes vs. no)	2.12	1.05–4.25	0.035	Anemia (yes vs. no)	2.12	1.05–4.25	0.035
WBC (≥15 vs. <15 × 10^9^/L)	0.47	0.21–1.05	0.067	WBC (≥15 vs. <15 × 10^9^/L)	0.47	0.21–1.05	0.067
Sβ_2_m (>2.0 vs. ≤2 mg/L)	not	selected	-	Sβ_2_m (>2.4 vs. ≤2.4 mg/L)	not	selected	-

WBC = White Blood Cell count * Variables examined in the multivariate model for all 915 patients: age (≥45 vs. <45 years), gender (male vs. female), stage (IV vs. IIB/III vs. I/IIA), B-symptoms (yes vs. no), infradiaphragmatic disease (yes vs. no), albumin (≥4 vs. <4 g/dL), leukocytosis (≥10 vs. <10 × 10^9^/L), anemia (yes vs. no), involved nodal sites (≥3 vs. <3), lymphopenia (yes vs. no), ESR (≥50 vs. <50 mm/h), and Sβ_2_m levels (>2.0 vs. ≤2 mg/L) or Sβ_2_m levels (>2.4 vs. ≤2.4 mg/L). ** Variables examined in the multivariate model for early-stage patients: age (≥45 vs. <45 years), gender (male vs. female), stage (II vs. I), leukocytosis (≥10 vs. <10 × 10^9^/L), anemia (yes vs. no), involved nodal sites (≥3 vs. <3), ESR (≥50 vs. <50 mm/h), and Sβ_2_m levels (>2.0 vs. ≤2 mg/L) or Sβ_2_m levels (>2.4 vs. ≤2.4 mg/L). *** Variables examined in the multivariate model for all advanced-stage patients: age (≥45 vs. <45 years), gender (male vs. female), stage (IV vs. IB/IIB/III), albumin (≥4 vs. <4 g/dL), marked leukocytosis (≥15 vs. <15 × 10^9^/L), anemia (yes vs. no), lymphopenia (yes vs. no), and Sβ_2_m levels (>2.0 vs. ≤2 mg/L) or Sβ_2_m levels (>2.4 vs. ≤2.4 mg/L).

**Table 5:** Summary of published studies on the prognostic significance of serum β_2_-microglobulin levels in patients with Hodgkin lymphoma.

Study	No. of Patients	Treatment		Prognostic Significance of β_2_-Microglobulin in Multivariate Analysis					
				Early Stages		Advanced Stages		Overall	
			Cutoff	PFS/TTF	OS	PFS/TTF	OS	PFS/TTF	OS
Oza et al., 1992 [65]	60 (IIIB, IV)	MVPP ± RT ChlvPP ± RT	3 mg/L	NA	NA	+ ^¶^	-	NA	NA
Dimopoulos et al., 1993 [48]	160	RT only NOVP ± RT, MOPP ± RT Anthracycline-based (minority)	2.5 mg/L	± *^,¶^	NT	+ *^,¶¶^	NT	+ *	NT
Fleury et al., 1994 [49]	64 (age < 50 y)	MOPP ± RT MOPP/ABVD ± RT	2.4 mg/L	NT	NT	NT	NT	+	NT
Axdorphet al., 2000 [64]	99	RT only MOPP or CCNU-OPP MOPP/ABVD ± RT	NR	NT	NT	NT	NT	- ***	- ***
Raida et al., 2002 [50]	69	NR	NR	NR	NR	NR	NR	- *	NR
Chronowski et al., 2002 [51]	191 (ES)	NOVP + RT, MOPP + RT ABVD + RT CVPP/ABDIC + RT	2.5 mg/L	± **	+	NA	NA	NA	NA
Visco et al., 2004 [66]	61 (ES, non-X)	RT only	“elevated”	+ *	NT	NA	NA	NA	NA
Vassilakopoulos et al., 2005 [53]	379	ABVD or equivalents ± RT	2.4 mg/L	+	+	-	-	- *	+
Itoh et al., 2010 [67]	167 (111)^ §^	ABVd ^§^ ± RT	2.0 mg/L	NT	NR	NT	NR	NT	-
Nakajima et al., 2014 [55]	67	ABVD ± RT	2.5 mg/L ^§§^	NR	NR	NR	NR	+	-
Wang et al., 2016 [56]	202 (IIX, III/IV)	ABVD ± RT	2.5 mg/L ^§§^	NA	NA	+ *	+	NA	NA
Miriliet al., 2019 [68]	122	RT only ABVD ±RT	2.2 mg/L ^§^	NT	NT	NT	NT	-	+
Wen et al., 2022 [69]	365	ABVD or equivalents ± RT	1.85 mg/L ^§^	NT	NT	NT	NT	-	+
Present Study, 2023	915	ABVD or equivalents ± RT	2.0 mg/L	+ *	-	± *	+	+ *	+

NA = Not Applicable, NT = Not Tested, NR = Not Reported, ES = early stage, non-X = non bulky. * Tumor control was the endpoint either as TTF or FFP (with progression/relapse counted as events along with treatment- or disease-related deaths or not; deaths of any other cause were censored). ** RFS was the endpoint (only recurrence counted as event). *** DFS and cause-specific survival were the endpoints. ^§^ 111/167 had sβ_2_m levels available; ABVd = ABVD with reduced dacarbazine doses. ^§§^ Cutoff determined by ROC curve analysis. ^¶^ Independent prognostic factor for achievement of CR but not for disease-free survival. ^¶¶^ The effect of sβ_2_m within early and advanced stages was tested only in univariate analysis.

## Data Availability

The data presented in this study are available on request from the corresponding author.
